# Cheminformatics to Characterize Pharmacologically Active Natural Products

**DOI:** 10.3390/biom10111566

**Published:** 2020-11-17

**Authors:** José L. Medina-Franco, Fernanda I. Saldívar-González

**Affiliations:** DIFACQUIM Research Group, Department of Pharmacy, School of Chemistry, Universidad Nacional Autónoma de México, Avenida Universidad 3000, Mexico City 04510, Mexico; fer.saldivarg@gmail.com

**Keywords:** ADME/Tox, chemoinformatics, chemical space, databases, drug discovery, molecular modeling, natural products, toxicity, web servers

## Abstract

Natural products have a significant role in drug discovery. Natural products have distinctive chemical structures that have contributed to identifying and developing drugs for different therapeutic areas. Moreover, natural products are significant sources of inspiration or starting points to develop new therapeutic agents. Natural products such as peptides and macrocycles, and other compounds with unique features represent attractive sources to address complex diseases. Computational approaches that use chemoinformatics and molecular modeling methods contribute to speed up natural product-based drug discovery. Several research groups have recently used computational methodologies to organize data, interpret results, generate and test hypotheses, filter large chemical databases before the experimental screening, and design experiments. This review discusses a broad range of chemoinformatics applications to support natural product-based drug discovery. We emphasize profiling natural product data sets in terms of diversity; complexity; acid/base; absorption, distribution, metabolism, excretion, and toxicity (ADME/Tox) properties; and fragment analysis. Novel techniques for the visual representation of the chemical space are also discussed.

## 1. Introduction

**Figure 1:**
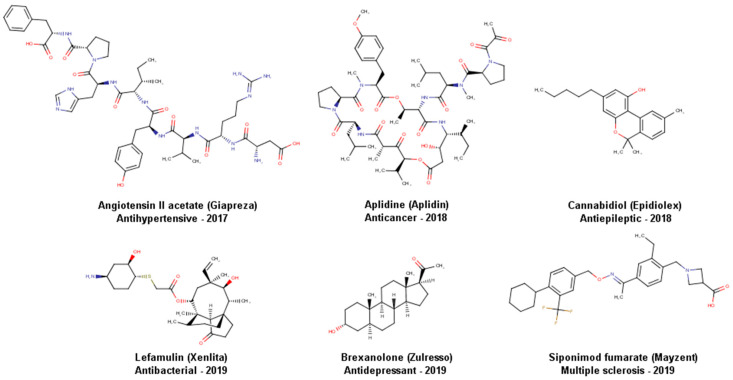
Recent natural products and derivatives approved for clinical use.

Natural products (NPs), from either terrestrial or aquatic organisms, have a long tradition as sources of active compounds for health-related benefits. From the approved drugs between 1981 and 2019, 3.8% corresponds to unaltered NPs, and 18.9% are NP derivatives [1]. Examples of unaltered NPs recently approved for clinical use are (Figure 1) angiotensin II acetate approved by the US Food and Drug Administration (FDA) in 2017 as a vasoconstrictor to increase blood pressure in adults with septic or other distributive shock [2]; aplidine, a new marine-derived anticancer agent approved for the first time in 2018 in Australia for the treatment of multiple myeloma [3]; and cannabidiol, approved in 2018 by the FDA as an antiepileptic drug [4]. Regarding NP derivatives, in 2019 the FDA approved nine drugs derived from NPs; among them are lefamulin used as an antibiotic, brexanolone an anti-depressant, and siponimod fumarate used to treat multiple sclerosis [1]. These compounds are illustrated in Figure 1. It is also well known that over millions of years, nature has selected and optimized chemical structures to produce chemical scaffolds and compounds enriched with biological function. However, NP hurdles include challenges in the isolation and purification procedures, minimal available amounts of lead compounds, the difficulty to synthesize NPs with high structural complexity, and issues associated with synthesis scale-up. Moreover, for drug discovery applications, caution should be taken with compounds that have been designed by nature for defense and are toxic. As such, one can expect that not all NP have a beneficial effect on health. However, the considerable success of using NPs to produce bioactive compounds or bioactive mixtures has inspired the preparation of synthetic molecules that have become drugs approved for clinical use [1]. Besides, the unique structural features of NPs, such as structural complexity [5], represent a promising opportunity to identify active or selective compounds for emerging targets [6] or those targets that are difficult to tackle with classical synthetic molecules.

The number of applications of computational approaches to improve and accelerate NP-based drug discovery is increasing. This fact is documented in several recent book chapters and review papers [7,8,9,10] that discuss the range of molecular modeling, chemoinformatics [11], and machine-learning approaches [12] that are used to elucidate, identify, and optimize drug candidates from natural origin, to understand the coverage of NP in chemical space, as well as prediction of bioactivity spectra, ADME (absorption, distribution, metabolism, and excretion) and safety profiles (toxicity), and in the quantification of natural product-likeness for natural product-inspired de novo design. Representative examples of studies in which computational methods for the identification of bioactive NPs were successfully used have been recently described by Chen and Kirchmair [10]. To name an example, in a pharmacophore-based virtual screening study, 14 NPs were identified as activators of the G protein-coupled bile acid receptor 1 (GPBAR1). Among these 14 NPs, two compounds, farnesiferol B and microlobidene (both compounds with reported EC_50_ values of approximately 14 µM) are based on molecular scaffolds that had not yet been associated with GPBAR1, which highlights the importance of NPs as sources of novel scaffolds for the development of chemical leads and innovative drugs [13].

This review aims to discuss recent applications of chemoinformatics methods to characterize in-depth NP data sets contents with potential therapeutic applications. We also present computational techniques to anticipate potential issues of toxicity. The review is organized into three sections. After this brief introduction of natural product-based drug discovery, Section 2 discusses recent progress on NP databases, emphasizing collections available in the public domain. The next section that is the core of the review, describes the NPs’ characterization and profiling in terms of physicochemical properties, molecular scaffolds, molecular complexity, fragments, acid/base and ADME/Tox profiling, global diversity, and visual representation of the chemical space. The last section presents conclusions.

## 2. Natural Product Databases

Compound databases have a prominent role in drug discovery. This is particularly relevant with the advent of big data. Indeed, high-throughput experimental and virtual screening of large chemical databases generates an enormous amount of data that need to be stored and made accessible to convert data into information and, finally, to knowledge [14]. One of the applications of chemoinformatics (also known as chem(i)oinformatics) in NP-research is the organization, analysis, and dissemination of chemical information of NP in compound databases [11,12]. There are several excellent and extensive reviews of NP databases, published over the past five years [9,15,16,17]. One of the most recent reviews is a compilation of more than 100 public NP databases from different sources that collect more than 400,000 non-redundant molecules [18]. Among the numerous NP databases in the public domain, there are initiatives to compile NPs from different geographical regions including Africa (e.g., African Natural Products Database—ANPDB) [19] and Latin America (e.g., Latin American Natural Product Database—LANaPD) [20] in a single platform.

Besides, there are efforts to make databases of fragments derived from NPs for NP-based fragment-based drug discovery and the generation of “pseudo-NPs” publicly available [21]. For instance, Chávez-Hernández et al. recently reported a large fragment library with nearly 206,000 fragments derived from a drug-like subset of the Collection of Open Natural Products (COCONUT) database. In that work, the fragment library of NP was compared to fragment libraries of ChEMBL, as representative of biologically relevant compounds, and a vast on-demand database of synthetic molecules. The fragment library of NPs was made freely available [22].

## 3. Chemoinformatic Profiling

In addition to the assembly and maintenance of NP databases, computational methods are used to analyze the compound databases’ contents and obtain a detailed profile of various features of common interest for drug discovery applications. Typical examples include the systematic analysis of chemical diversity using different structural and molecular representations, a profile of physicochemical properties of pharmaceutical interest, molecular complexity, visual representation of the chemical space, and in silico profiling of absorption, distribution, metabolism, excretion, and toxicity (ADME/Tox). There are well-established chemoinformatic protocols to obtain a detailed profile of these characteristics [23]. Table 1 summarizes examples of recent chemoinformatic profiling of compound databases, which are discussed in the following sections.

### 3.1. Physicochemical Properties

Molecular descriptors frequently used to describe chemical libraries include molecular weight (MW), the octanol/water partition coefficient (SlogP), topological polar surface area (TPSA), hydrogen bond donors (HBD), hydrogen bond acceptors (HBA), and the number of rotatable bonds (RB). These descriptors are typically used to quantify lead-like and drug-like features of compound data sets. In general, these descriptors are intended to capture three significant features of interest in drug development, namely size (MW), polarity (SlogP, TPSA, HBDs, HBAs), and flexibility (RBs) [35,36]. NP data sets have been profiled for more than 15 years with these six molecular descriptors. It is also common to include in such analysis the distribution of other basic yet relevant structural features such as counts of carbon, nitrogen, oxygen atoms, and different types of rings (total number, aromatic, heteroaromatic, etc.) [37,38].

Examples of recent profiling of physicochemical properties of NP data sets are summarized in Table 1. Pilón-Jimenez et al. did a comparative analysis of BIOFACQUIM, a NP database from Mexico with drugs approved for clinical use, NPs from NuBBE_DB_, marine NPs, cyanobacteria, and fungi metabolites [39]. Authors of that work concluded that compounds in BIOFACQUIM are more similar to NuBBE_DB_ and fungi data sets. In a separate and also recent study, Simoben et al. reported the drug-likeness of 1870 compounds from the EANPDB database (Table 1) [19]. It was found that about 85% of the compounds in this database have drug-like features.

In 2020, Saldívar-González reported a diversity analysis based on physicochemical properties of 154,680 compounds from the Universal Natural Product Database [23] and compared its diversity to compound data sets from different origin such as 188 morpholine peptidomimetics from a diversity-oriented-synthesis (DOS) approach, 37 analogs of indinavir from a combinatorial library, 27 non-nucleoside DNA-methyltransferase inhibitors from a lead optimization program (representative of a target-oriented synthesis approach, TOS), and drugs approved for clinical use. The authors concluded that compounds from the extensive NP database are the most diverse, while compounds from the combinatorial library, followed by the TOS set, are the least diverse.

### 3.2. Molecular Scaffolds

Molecular scaffolds, also termed “chemotypes” are the main or core of a molecular structure. Like physicochemical properties discussed in Section 3.1, molecular scaffolds are straightforward to interpret and facilitate communication across different disciplines such as NP and medicinal chemists, and chemoinformaticians. Certainly, molecular scaffolds are firmly bound to general concepts in drug discovery, such as “privileged structures” [40] and “scaffold hopping” [41]. There are different ways to generate scaffolds of compound databases systematically and consistently that have been extensively reviewed by Langdon [42].

Systematic analysis of NP databases’ scaffold content has been reported revealing the most frequent and distinct scaffolds in the data sets. For instance, Saldívar-González et al. identified the most common scaffolds found in NPs from Brazilian diversity [27]. Tran et al. reported the unique molecular scaffolds present in compounds from honey bee and stingless bee propolis (Table 1). In the same study, authors readily identified that benzene, coumarin, flavane, and flavone are the four scaffolds present in the propolis plus approved drugs and food chemicals [31]. Al Sharie et al. analyzed the scaffold diversity of metabolites from red, brown, and green algae from the Seaweed Metabolite Database, concluding that red algae metabolites are the least diverse while metabolites from green algae are the most diverse [32]. Similarly, González-Medina et al. also recently analyzed the scaffold diversity of cyanobacteria compounds from freshwater and marine sources, concluding that the former are less diverse than metabolites from marine sources. In that work, the most frequent scaffolds found in both data sets and the molecular scaffold common to both compound collections were also revealed [26].

**Figure 2:**
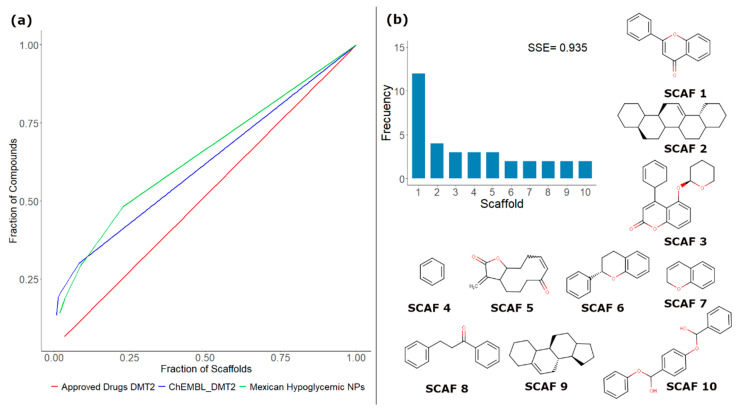
(**a**) Cyclic system retrieval (CSR) curves for three different libraries: natural products (NPs) isolated from Mexican hypoglycemic plants [32] (green), drugs approved to treat diabetes mellitus type 2 (DMT2) (red), and compounds evaluated to treat DMT2 deposited in the ChEMBL (blue); (**b**) Distribution and Shannon entropy for the 10 most frequent chemotypes in active NPs isolated from Mexican hypoglycemic plants.

To illustrate the concept of scaffold content and diversity, Figure 2a shows a Scaffold Recovery Curve (CSR), which directly compares the content and diversity of scaffolds from three different databases. For this example, 86 active NPs isolated from Mexican hypoglycemic plants [43], 30 drugs approved to treat diabetes mellitus type 2 (DMT2), and 193 compounds evaluated to treat DMT2 deposited in the ChEMBL database were compared. Scaffolds were generated under the Bemis–Murcko definition [44]. As can be seen in Figure 2a, the database of drugs approved to treat DMT2 is the one that contains the largest diversity of scaffolds. This is reflected in the fact that its line resembles a diagonal, which means that each compound in the database has a different scaffold. In contrast, in this example, the curves of NP isolated from Mexican hypoglycemic plants and the compounds obtained from ChEMBL evaluated to treat T2DM show an increase in their slope, indicating that these data sets have a lower diversity of scaffolds. Quantitatively, the CSR curves can be compared using two metrics: area under the curve (AUC) and the fraction of chemotypes that recover 50% of the molecules in the data set (F_50_). Based on these metrics, the diversity order decreases in the following order: approved drugs for DMT2 (AUC = 0.515) > compounds from ChEMBL evaluated for DMT2 (AUC = 0.615) > Mexican hypoglycemic NPs (AUC = 0.645). For a more comprehensive analysis of the scaffold diversity, another frequently used metric is Shannon’s entropy (SE) [45]. Unlike the CSR curves that quantify the diversity of the entire data sets, SE has been employed to measure the scaffold diversity of the most populated scaffolds. To normalize SE to the different numbers of scaffolds, n, it is common to use the Scaled Shannon Entropy (SSE). The SSE value oscillates between 0 when all the compounds have the same chemotype (minimum diversity) and 1.0 when all the compounds are evenly distributed among the n acyclic and/or cyclic systems (maximum diversity). To illustrate this concept, Figure 2b shows SSE for the ten most frequent scaffolds in the database of NPs isolated from Mexican hypoglycemic plants. In this case, an SSE10 value of 0.935 was obtained, and the most frequently predominant scaffold is flavone (SCAF 1).

### 3.3. Molecular Complexity

Similar to molecular similarity [46], molecular complexity is an ambiguous and subjective concept which definition depends on the person’s experience and application. For instance, the complexity of a molecule can be assessed in terms of the final structure itself (atom connectivity or three-dimensional shape) or how difficult it is to synthesize. There are metrics that have been proposed to quantify the complexity of the structure of a molecule [5]. Likewise, there are different approaches to measure synthetic accessibility [47]. Saldívar-González et al. recently reviewed the three main methods to evaluate chemical complexity and synthetic accessibility, namely graph-theoretical methods, (sub)structure-based approaches, and physicochemical and topological descriptors [23]. In that review, it is noted that the results of the quantitative metrics should coincide with the chemical intuition. Likewise, it is emphasized that simple metrics can provide insightful results [23].

Quantitative assessment of molecular complexity is becoming a crucial factor in drug discovery since it has been associated with increased probabilities to advance in clinical development [48], selectivity, and safety. Recently, it has been proposed that a classical metric to quantity structural complexity such as the fraction of sp^3^ carbon atoms (Fsp^3^) is a drug-likeness criterion [49]. Several metrics are straightforward to compute with open-source and free software such as DataWarrior [50,51].

It is well known that NPs can have highly complex structures. Likewise, several NPs’ synthetic accessibility is difficult, particularly when they have several stereocenters. Chemoinformatic methods are useful for quantifying the molecular complexity and comparing it with the complexity of compounds from other sources such as organic synthesis. Over the past few years, Fsp^3^, the number of stereocenters, and other descriptors have been used to compare the molecular complexity of NPs from different sources and geographical regions. Prieto-Martínez et al. recently reviewed several analyses [8]. More recent studies include the Universal Natural Products Database’s molecular complexity profiling, NPs from Brazil collected in NuBBE_DB_, marine NPs, cyanobacteria, fungi metabolites, and other data sets [27]. It has also been recently analyzed the complexity of compounds from the Seaweed Metabolite Database [32] (Table 1). It has been concluded that, overall, NPs are more complex than drugs approved for clinical use and that NPs have large differences in complexity, depending on the particular source. For instance, cyanobacterial metabolites are more complex than fungi metabolites. Moreover, marine metabolites are more complex than NPs available from commercial sources [27].

### 3.4. Fragments

The overall complex chemical structures of NPs make them attractive sources to investigate novel areas of chemical space. Simultaneously, high structural complexity represents a challenge to further obtain them in large quantities needed in advance stages of the drug development face. For this reason, there has been a recent interest in developing synthetic plans to generate semi-synthetic compound libraries inspired by NPs [52]. Moreover, NPs are attractive starting points for fragment-based drug design and generating “pseudo-NPs” [21]. Based on the need to generate fragment libraries based on NPs, Chávez-Hernández et al. recently reported an exhaustive library with 205,903 fragments obtained from a sizeable drug-like subset of COCONUT (vide supra) [22]. In that work, the authors compared the NP-based fragment collection with a fragment library obtained from more than 1 million drug-like compounds tested for biological activity and stored in ChEMBL [53], and with a second fragment collection derived from more than 15 million synthetically accessible and novel compounds. It was concluded that there is an extensive diversity of unique fragments derived from NPs that could be used as building blocks for the de novo design and synthesis of unique molecules. It was also found that the entire structures and fragments derived from NPs are more diverse and have larger structural complexity than the two reference compound collections [22].

### 3.5. Acid and Base Profiling

Acidic and basic functional groups of a molecule determine its charge state at different pH values. This, in turn, can affect its solubility, physicochemical properties, affinity for a molecular receptor, pharmacokinetics, and toxicity (vide infra). For instance, molecular basicity has been correlated with molecular promiscuity, hERG blockade, and phospholipidosis. The reader is directed to an in-depth analysis by Manallack et al. [54] on the effect of acid/base properties on ADME/Tox properties, drug–target interaction, and drug formulation.

Despite the critical importance of the acid/based properties of molecules in drug discovery, they have been analyzed on a limited basis for NPs. Just recently, Santibáñez-Morán et al. discussed the acid/base profile of NP libraries from different geographic locations and sources. The calculated profile was compared to food chemicals and drugs approved for clinical use [28,55]. The NP data sets analyzed were the Universal Natural Product Database, NPs from NuBBE_DB_ and BIOFACQUIM databases, marine NPs, fungi and cyanobacteria metabolites, and NPs from commercial vendors (pure and semi-synthetic). The NP data sets were compared to food chemicals and drugs approved for clinical use (Table 1). Santibáñez-Morán et al. concluded that, regardless of the different characteristics of the various NP data sets depending on the source of origin (marine, fungi, cyanobacteria) and geographical location (e.g., Brazil, Mexico), NPs contain about 45% of neutral compounds. NP also have around 25% of single acids with a pK_a_ distribution comparable to approved drugs and less than 7% of single bases.

### 3.6. ADME/Tox Profiling

ADME/Tox properties have a significant role in drug discovery [56]. It is estimated that around 40% of all drug failures are related to issues with such properties. Therefore, early measurement or at least in silico prediction of ADME/Tox properties has a large impact on drug development projects. However, accurate prediction of such properties is not a trivial endeavor, but big data and machine learning are largely contributing to improving ADME/Tox predictions [57,58]. A large variety of prediction methods have been implemented into public web servers [59]. For instance, Jia et al. reviewed public online resources to evaluate the ADME and drug-likeness properties of compound datasets [56]. The authors emphasized that quality and updated information in comprehensive databases are key factors for constructing reliable models to evaluate drug-likeness in silico. Jia et al. also concluded that online ADME/Tox resources provide useful guidelines to extract rational compounds that match the desirable pharmacokinetic properties or to filter compounds that are not likely to be drugs. Chen et al. have pointed out that, despite the fact there are several web servers and computational models of free access to evaluate ADME/Tox properties, the user should be careful as many of such models have been trained on synthetic compounds, and the applicability domain of NPs could be outside those models [10].

Since NPs are excellent sources of drug candidates, NP data sets have been profiled for the past 15 years [60]. For instance, Fatima et al. recently discussed a computational ADME/Tox profiling of four phytochemical databases (Phytochemica, SerpentinaDB, SANCDB, and NuBBE_DB_) covering the regions of India, Brazil, and South Africa, analyzing different parameters. The authors concluded that 24 compounds belonging to five chemical classes (alkaloids, flavonoids, terpenes, lignoids, and phenols) and 15 different plant sources have the ADME/Tox properties that can be considered for drug development [33].

Durán-Iturbide et al. reported a comparative in silico profile of compounds in BIOFACQUIM with NP from AfroDB, NuBBE_DB_, molecules from the Traditional Chinese Medicine (TCM), and drugs approved for clinical use. Authors of that work found that the absorption and distribution profile of compounds in BIOFACQUIM is similar to approved drugs, while the metabolism profile is comparable to other NP databases. The excretion profile of compounds in BIOFACQUIM is different from approved drugs, but their predicted toxicity profile is comparable [30].

Simoben et al. reported the ADME/Tox profile of 1870 compounds from the EANPDB database (Table 1) [19]. To that end, the authors employed the free-server pkCSM-pharmacokinetics [61]. It was found that 99.7% of the molecules in EANPDB were predicted to do not interfere with the inhibition of the potassium ion (K^+^) channels. It was also estimated that about 85% of compounds in EANPDB do not have hepatotoxic or skin sensitization effects [19].

Currently, new multi-objective models built by artificial neural networks and based on methods like Perturbation Theory ML (PTML) are used to correctly predict biological activity, toxicity, ADME properties and classify compounds under experimental conditions [62]. These methods were successfully applied in various studies. For example, Speck-Planchee et al. [63] further proposed the first mtk-QSBER model to simultaneously explore antibacterial activity against Gram-negative pathogens and in vitro safety related to absorption, distribution, metabolism, elimination, and toxicity (ADMET). The accuracy of this model in both the training and prediction (test) sets is higher than 97%. They also have developed a chemoinformatic model for the simultaneous prediction of anti-cocci activities and in vitro safety [64]. These types of models represent a promising field for the study of NPs.

### 3.7. Global Diversity

As commented on in the previous sections, different representations of chemical structures (physicochemical properties, sub-structural features, molecular fingerprints, etc.) are used to measure the chemical diversity of compound data sets. Indeed, chemical representation is one (or perhaps the most important) feature in chemoinformatics (vide infra). Therefore, molecular diversity is highly attached to the particular method used to quantify diversity. To reduce molecular diversity dependence with molecular representation has been proposed to combine multiple representations into a single graph termed Consensus Diversity Plot (CDP) [65]. A CDP is a bi-dimensional graph that shows on the same plot four measures of diversity (more metrics of diversity could be added), and it is intended to evaluate the “total” or “global” diversity of compound data sets. In current applications of CDPs, the most common representations to analyze diversity have been scaffold-based, fingerprint, drug-like molecular properties, and the number of compounds (or size) in the data set. Complexity has also been represented. There is a free webserver to generate CDPs [65].

CDPs were used to analyze the total diversity of NPs from Brazil, Mexico, and Panama [23,26,28]. Recently Al Sharie et al. employed the consensus technique to compare metabolites from red, brown, and green algae from the Seaweed Metabolite Database, concluding that metabolites from green algae are the most diverse, overall [32]. The graphs have also been used to analyze the global diversity of compounds tested with epigenetic targets and synthetic libraries [66]. Further discussion of CDP has been published recently [23].

### 3.8. Chemical Space: Visual Representation

The concept of “chemical space” has received different definitions. For instance, Virshup et al. define this concept as “an M-dimensional Cartesian space in which compounds are located by a set of M physiochemical and/or chemoinformatic descriptors” [67]. Such a definition emphasizes the dependence of chemical space with molecular representation. Although many quantitative assessments of the structural diversity of compound data sets are linked to the concept of chemical space (e.g., analysis of the profile of the six physicochemical properties of pharmaceutical interest, vide supra), the chemical space exploration is usually associated with a visual representation of the multi-dimensional space. To this end, different visualization techniques have been implemented. Amongst the most common are principal component analysis (PCA), self-organizing maps (SOM), t-distributed stochastic neighbor embedding (t-SNE), ChemMaps [68], and others extensively reviewed in [69,70]. Another representation that uses molecular descriptors is the Principal Moment of Inertia (PMI) plot, which represents the shape distribution of the molecules in a library [71]. For example, Olmedo et al. used PCA to generate a comparative chemical space visualization of NPs from Panama with compounds in TCM, synthetic molecules, and drugs approved for clinical use [24,25].

Recently, Probst proposed the technique Tree Map (TMAP) tuned to visualize high-dimensional chemical spaces [72]. This technique was used to visualize the chemical space of the NP database BIOFACQUIM with the reference databases ChEMBL and NP assembled from the Universal Natural Products Database, the Natural Products Atlas, and Natural Products in PubChem Substance Database [29]. Visual representation of the chemical space is often used to systematically explore the structure-activity relationships (SAR) or structure multiple-activity relationships (SMARts) [73] of compound data sets and identify valuable “StARs” (structure-activity relationships) in chemical space [74].

The chemical space of NPs from plant, marine, fungi, and other sources was extensively reviewed by Saldívar-González et al. [75]. In that review, the authors highlight the variety of properties calculated and different visualization methods of the chemical space. The molecular representations used more frequently to visualize the chemical space are physicochemical properties associated with drug-like features and molecular fingerprints. One of the most frequently used visualization techniques is PCA. In that work, it was concluded that the space of naturally occurring molecules is diverse and vast. The consistent exploration of the space may have crucial implications not only in drug discovery but also in biodiversity analysis.

In a novel approach, Santibáñez-Morán et al. reported a PCA representation of chemicals from seven NP data sets of different origin (e.g., marine, fungi, and cyanobacteria metabolites) and geographical region (Brazil and Mexico) using nine descriptors associated to the acid/based profile [28]. The NP data sets were compared to food chemicals and drugs approved for clinical use. The first two principal components captured 76% of the variance. The visualization of the chemical space and hierarchical clustering of the same nine descriptors revealed that cyanobacteria metabolites are different from the other NP data sets due to mainly the different pKa distribution of single acids that, in turn, is associated to the low proportion of carboxylic acids. The analysis also showed that a commercial’s vendor semi-synthetic compounds data set is more similar to drugs approved for clinical use [28].

Sánchez-Cruz et al. used the TMAP method to visualize the chemical space of 503 compounds in BIOFACQUIM, 168,030 NPs assembled from three large data sets (the Natural Product Atlas, Natural Products in PubChem Substance Database, and Universal Natural Product Database), and 1,667,509 compounds from ChEMBL 25 [29]. TMAP was particularly useful in this case since, as stated above, this approach is suitable to represent visually large data sets as a two-dimensional tree. It was found that compounds in ChEMBL practically defined the biologically relevant chemical space, but this is not evenly populated. In such reference space defined by ChEMBL, NPs cover the same space but more sparsely. In contrast, compounds in BIOFACQUIM populate less dense chemical space regions but have compounds similar to the reference data sets [29].

**Figure 3:**
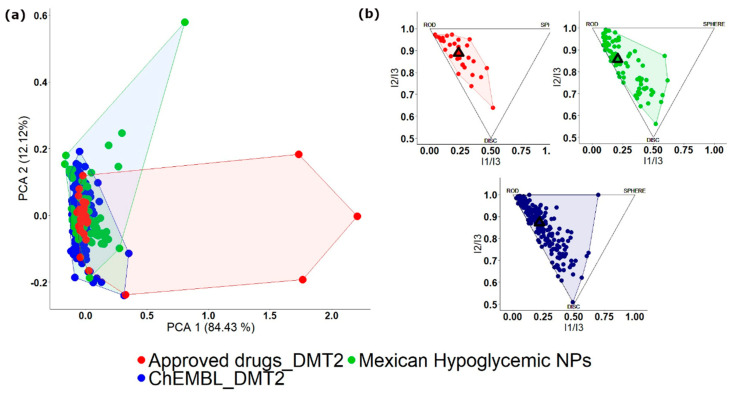
Visual representation of the chemical space of three different libraries: NPs isolated from Mexican hypoglycemic plants (green), drugs approved to treat DMT2 (red) and compounds from ChEMBL evaluated to treat DMT2 (blue) (**a**) Principal Component Analysis (PCA) plot generated using six structural and physicochemical descriptors (molecular weight, hydrogen bond donors, hydrogen bond acceptors, the octanol and/or water partition coefficient, topological polar surface area, and number of rotatable bonds); (**b**) Principal Moments of Inertia (PMI) plot. Compounds are placed in a triangle where the vertices represent rod, disc, and spherical compounds.

To illustrate a visual representation of the property-based chemical space, Figure 3 depicts the comparison of the three libraries analyzed in a previous example (Section 3.2). Figure 3a shows the PCA of 86 active NPs isolated from Mexican hypoglycemic plants [43], 30 drugs approved to treat DMT2, and 193 compounds evaluated to treat DMT2 deposited in the ChEMBL. In this figure, the database of drugs approved to treat DMT2 covers most of the physicochemical chemical space followed by the database of NPs isolated from Mexican hypoglycemic plants. Regarding the diversity of shapes, as observed in the PMI plot (Figure 3b), the ChEMBL compounds evaluated to treat DMT2 are those that contain the largest diversity of shapes, followed by NPs isolated from Mexican hypoglycemic plants. In contrast, the base of drugs approved to treat DMT2 is the one that presents the least diversity when the shape of its compounds is evaluated. In both representations, it is observed that most NPs isolated from Mexican hypoglycemic plants share chemical space with approved drugs and compounds evaluated to treat DMT2; therefore, they represent an interesting source for the design of new hypoglycemic compounds.

## 4. Conclusions

Chemoinformatics is crucial for organizing and maintaining the information of NPs in chemical databases. Likewise, chemoinformatic approaches enable us to perform many comparative analyses of NP databases of different geographical regions or sources with synthetic compound libraries and drugs approved for clinical use. Such analyses quantitatively show NPs’ unique characteristics, such as molecular complexity and acid/base profile. Chemoinformatic analyses also reveal that NPs may have significantly different chemical structures, depending on the source. For instance, cyanobacteria metabolites have a remarkably different physicochemical properties profile compared to fungi metabolites from plants. It is also concluded that molecular representation has a profound impact on the analysis’ use and interpretation. For instance, the visual representation of the chemical space of NPs largely depends on the molecular descriptors.

In the past few years, NP databases and in silico models to analyze such databases’ diversity and predict properties such as ADME/Tox characteristics and other properties of pharmaceutical relevance have been implemented on free online servers. This facilitates the rapid access to quality data and performing rapid cross-comparisons of chemical information.

Other relevant chemoinformatics applications to NP-based research such as computer-aided NP selection, identification of molecular targets for NPs, de novo design, and quantification of NP-likeness have been reviewed recently by Chen et al. [10]. This review paper provides additional insights into the broad scope of chemoinformatics to NP research, in particular with an emphasis on drug discovery. The study of NPs still poses some extraordinary challenges. However, it is anticipated that as more quality data is available in NP research, such as biological activity data, cheminformatics will integrate new algorithms and machine learning techniques to accelerate NP-based drug discovery.

## Figures and Tables

**Table 1:** Examples of recent chemoinformatic profiling of natural products (NPs) data sets.

Data Set	Goal and Approach	Reference
454 NP from Panama.	Build and characterize the contents and diversity of a NP collection from Panama. Comparison with NP from other geographical regions.	[24,25]
560 cyanobacteria metabolites (freshwater and marine).	Quantify the distribution of drug-like properties; measure the diversity using properties, molecular fingerprints, and molecular scaffolds.	[26]
209,574 compounds from the Universal Natural Products Database and other NPs.	Comparative analysis of molecular complexity diversity based on physicochemical properties, molecular scaffolds and fingerprints. Comparison with drugs approved for clinical use.	[23,27]
209,574 compounds from the Universal Natural Products Database, 423 molecules from BIOFACQUIM and other NPs.	Comparative analysis of the acid/based profile of NP from different sources. Comparison with drugs approved for clinical use and food chemicals.	[28]
503 NPs from Mexico collected in the BIOFACQUIM database.	Diversity analysis based on different molecular representations and ADME/Tox profiling.	[29,30]
578 compounds from honey bee and stingless bee propolis.	Analysis of chemical space, chemical diversity, and scaffold content.	[31]
897 metabolites from the Seaweed Metabolite Database (SWMD).	Diversity analysis based on different molecular representations.	[32]
1870 compounds from the Eastern Africa Natural Product Database (EANPDB).	Quantification of scaffold diversity and profiling of drug-likeness and ADME/Tox properties.	[19]
NPs from four NP data sets: phytochemica, SerpentinaDB, SANCDB, and NuBBE_DB_.	In silico profiling of ADME/Tox properties.	[33]
6524 NPs originating from about 3300 producer streptomycetes strains	In addition to names and molecular structures of the compounds, information about source organisms, references, biological role, activities, synthesis routes, scaffolds, physicochemical properties, and predicted ADMET properties is included.	[34]
